# From neglect to equity in snakebite envenoming; what the ICMR-Collaborative Centre of Excellence (CCoE) targets

**DOI:** 10.1371/journal.pntd.0012425

**Published:** 2024-09-12

**Authors:** Jaideep C. Menon, Bipin Nair, Sanghamitra Pati, Vijay V. Pillay, Amarendra Mahapatra, T. P. Sreekrishnan, Muralidharan Vanuopadath, Denny John, Sabarish B. Nair, Prakash K. Sahoo, Aravind M. S., Aswathy Sreedevi, Chandrasekhar Jankiram, Joseph K. Joseph

**Affiliations:** 1 Professor, Adult Cardiology and Public Health, Amrita Institute of Medical Sciences, Amrita Vishwa Vidyapeetham, Kochi, India; 2 Professor & Dean, Amrita School of Biotechnology, Amrita Vishwa Vidyapeetham, Kollam, India; 3 Director, ICMR, Regional Medical Research Centre, Bhubaneswar, India; 4 Head, Poison Control Centre and Department of Forensic Medicine & Toxicology, Amrita Institute of Medical Sciences, Amrita Vishwa Vidyapeetham, Kochi, India; 5 Scientist G, ICMR, Regional Medical Research Centre, Bhubaneswar, India; 6 Consultant, Emergency Medicine & Critical Care, Amrita Institute of Medical Sciences, Amrita Vishwa Vidyapeetham, Kochi, India; 7 Assistant Professor, Amrita School of Biotechnology, Amrita Vishwa Vidyapeetham, Kollam, India; 8 Professor, Faculty of Life and Allied Health Sciences, Ramaiah University of Applied Sciences, Bengaluru, India; 9 Consultant, Emergency Medicine & Critical Care, Amrita Institute of Medical Sciences, Amrita Vishwa Vidyapeetham, Kochi, India; 10 Scientist D, ICMR, Regional Medical Research Centre, Bhubaneswar, India; 11 Research Associate, Department of Public Health, Amrita Institute of Medical Sciences, Amrita Vishwa Vidyapeetham, Kochi, India; 12 Professor & Head, Department of Community Medicine, Amrita Institute of Medical Sciences, Amrita Vishwa Vidyapeetham, Kochi, India; 13 Professor & Head, Public Health Dentistry, Amrita School of Dentistry, Amrita Vishwa Vidyapeetham, Kochi, India; 14 Senior Consultant Nephrologist, Little Flower Hospital and Research Centre, Angamaly, India; University Hospital, GERMANY

## Background

Among the neglected tropical diseases (NTDs), which affect a fifth of the world’s population and disproportionately affect underprivileged communities in the Tropics, venomous snakebite is arguably the most neglected. The World Health Organization’s (WHO) list of neglected diseases (NTDs) included snakebite envenoming (SBE) in 2009. In 2013, the condition was reclassified as a neglected one, and in 2015, the WHO delisted SBE. Following a passionate plea from the late Kofi Annan, the former UN Secretary-General, representatives from Médecins Sans Frontières (MSF), the Global Snakebite Initiative (GSI), the Health Action Initiative (HAI), and other stakeholders from member countries, it was subsequently reincluded as an NTD in 2017. Notably, SBE is responsible for more deaths than all other NTDs combined [1–3].

India is disproportionately affected by SBE in terms of both incidence and mortality. Based on WHO data, it is estimated that between 1.8 and 2.7 million envenoming results from between 4.5 and 5.4 million bites worldwide each year. Out of them, 81,000 to 138,000 victims die from complications, and an additional 400,000 suffer permanent disabilities [1]. It is estimated that 58,000 victims in India die from SBE each year, which accounts for half of all deaths worldwide [4]. This is in contrast to Australia and the United States, where there are equally venomous species present but the death toll is in the single digits [5]. Males account for over 70% of SBE cases, which usually occur in the productive ages range of 20 to 60 years. The socioeconomic effects of SBE can be disastrous, particularly with the death or disability of the sole earning member of the family. In rural communities, SBE also plays a major role in the loss of livestock [6].

## Story of neglect

SBE is a condition linked to poverty and impoverishment, which predominantly occurs in rural hinterlands. The fact that SBE is a significant cause of death in India reflects the inherent inequities and inequalities between urban and rural areas, genders, and states, keeping in mind that health is a state subject in India’s federal system of governance. Inevitably, the burden and complications of SBE are highest in areas with the least developed health and infrastructural facilities. Due to the lack of trained healthcare professionals available around the clock in Primary Health Centres (PHC), victims of snakebite must rely on alternate healers for the initial and immediate treatment of SBE. The fact that approximately 65% of bites are from nonvenomous or mildly venomous species of no medical significance, and that approximately half of bites from known venomous species are “dry,” leaves us with 20% to 30% of bites resulting in envenomation. These nonvenomous or dry bites provide alternative systems with an opportunity to thrive and establish a successful reputation as healers [7–10].

The WHO released a global strategy in 2019 to mitigate SBE. The strategy has 4 main subthemes: strengthening health systems; empowering and engaging communities; ensuring safe and effective treatment; and increasing partnerships, coordination, and resources. The WHO believes that these actions will help achieve the SDG (Sustainable Development Goal) target of reducing death and morbidity by 50% by the year 2030 [1,7,8].

The Government of India, through Ministry of Health and Family Welfare (MoHFW) announced the launch of a National Programme for SBE Prevention and Control in September 2022.

The Indian Council of Medical Research (ICMR) designated the Amrita Institute of Medical Sciences (AIMS) in Kochi as a Collaborating Centre of Excellence (CCoE) for snakebite in November 2023. Only a few centres worldwide are currently devoted to snakebite research: the Australian Venom Research Unit in Melbourne, the Liverpool School of Tropical Medicine, and the Snakebite Research and Intervention Centre in Kenya.

## CCoE; mandate and way forward

The CCoE at AIMS, Kochi is dedicated to the development and implementation of programmes that are consistent with the broad subthemes of the WHO strategy for the mitigation of SBE. The Centre of Excellence (CoE) is primarily intended to serve as a knowledge partner of the ICMR for the generation of evidence and as a coordinating unit for research among the various groups involved in SBE. Along with physicians, this would include ophiologists and herpetologists, snake rescuers, members of civil society and nongovernmental organisations focusing on community awareness and prevention, basic scientists conducting venom research, and government agencies such as Forest and Disaster boards. One of the primary roles envisaged is that of a resource and information bank in the field of SBE, with a dedicated website that updates on field activities via the CCoE or otherwise, recent developments, a map of hotspots, information on snakes, venom and snakebite, and so on. The CCoE would also collaborate with global organisations and networks working in the field of SBE, such as the WHO, MSF, GSI, and HAI.

**Fig 1:**
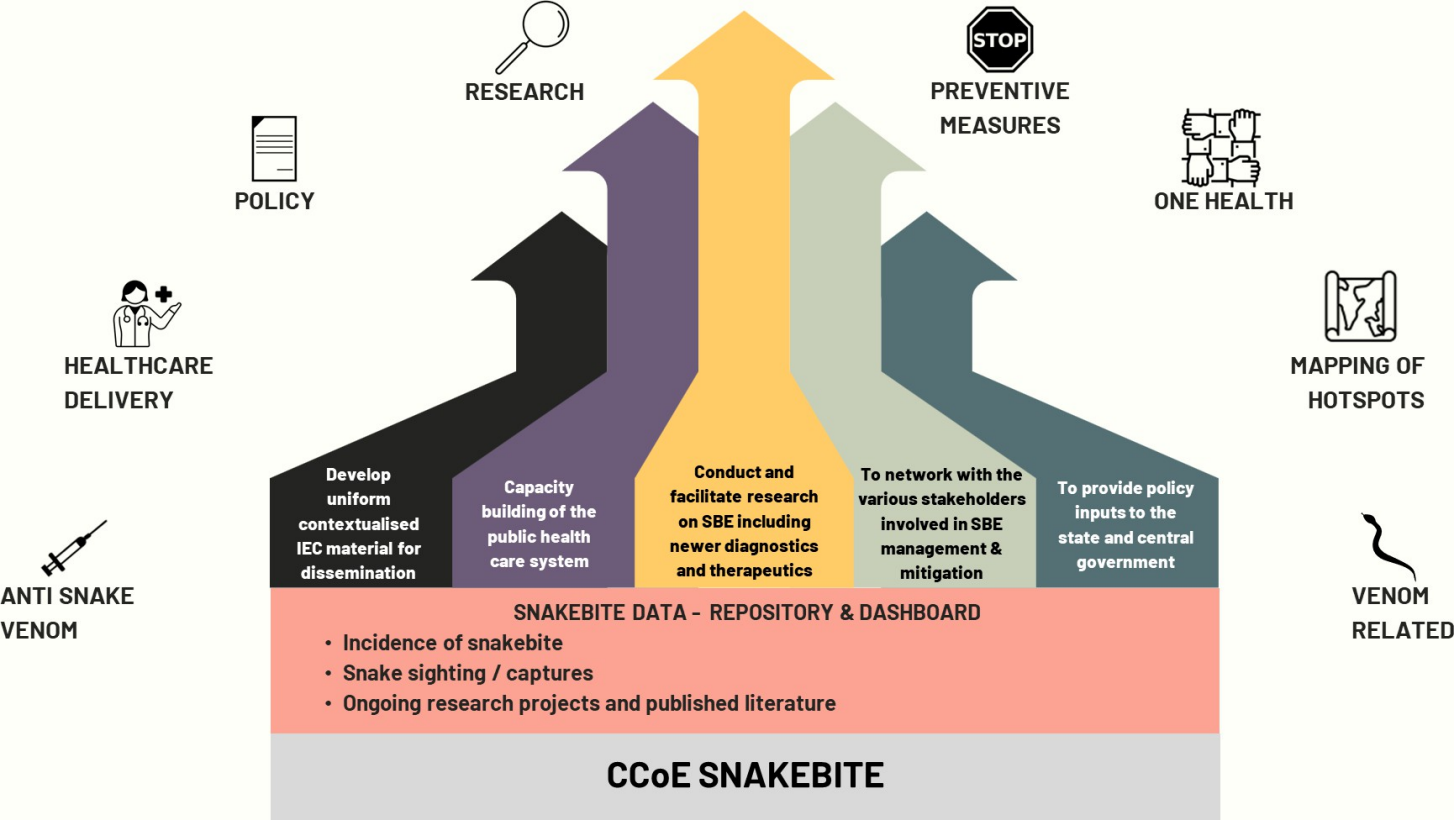
Illustrates the aspects of SBE that the CCoE hopes to address. (Icon credit and link to the licence, map: https://commons.wikimedia.org/wiki/File:Noun_Project_Map_icon_1463108.svg, hands: https://commons.wikimedia.org/wiki/File:Teamwork_icon_from_Noun_Project.png, magnifier: https://commons.wikimedia.org/wiki/File:Noun_Project_research_icon_4479310.svg, Doctor: https://commons.m.wikimedia.org/wiki/File:Noun-doctor-2909354-00449F.svg and stop: https://commons.wikimedia.org/wiki/File:Stop-sign_-_Delapouite_-_game-icons.svg Syringe; https://commons.wikimedia.org/wiki/File:Syringe_-_The_Noun_Project.svg,policy:https://commons.wikimedia.org/wiki/File:Policy_(50034)_-_The_Noun_Project.svg, Snake; https://commons.wikimedia.org/wiki/File:Snake_silhouette.svg).

The CCoE would strive to establish itself as a knowledge partner by generating evidence of best practices, fostering research, collaborating with other stakeholders in the field of SBE, conducting workshops and conferences on a variety of topics related to SBE, advocacy, community awareness, reviewing treatment protocols and circulating across the public health system, developing the template for easy to follow flow diagrams on first-aid measures, and treatment protocol, and signs and symptoms of envenoming (Fig 1).

The major issues related to SBE contributing to neglect have already been covered in a white paper (Chakma and colleagues) [9], as well as the challenges ahead and recommendations for the National snakebite prevention and control programme (Gajbhiye and colleagues, IJMR) [10].

Different aspects of the SBE issue that would be addressed include the following:

1. **Anti-Snake Venom (ASV)** [11,12]
  - Anti-sera against medically relevant snakes other than the Big 4 (the commercially available ASV in India covers the venom of the so-called Big 4 species: *Daboia russelii*, *Bungarus caeruleus*, *Naja naja*, and *Echis carinatus*)
  - Quality improvement of commercially available ASV
  - Assuring the availability of ASV in SBE hotspots
2. **Venom Related** [12,13]
  - Zonal venom pools (venom for the manufacture of ASV is primarily sourced from a single cooperative in South India)
  - Milking snakes in captivity
  - The “omics” approach to the study of venoms of all medically relevant species in India, along with thorough biochemical and pharmacological characterizations
3. **Healthcare system and delivery** [14,15]
  - Training of doctors, especially in hotspots, on the management of SBE
  - Training materials for PHCs regarding local venomous species, first aid protocols, and treatment guidelines
  - Training in life support skills is being provided to all MBBS doctors and specific targeted groups, including the fire force, police, drivers, flight cabin crew, health workers, forest guards, school children, and teachers
  - A mobile app for district and state mapping of all hospitals that treat SBE
  - Addressing the topic of SBE in both the Emergency Medicine and Internal Medicine textbooks
  - Guidelines for the management of snakebite will be widely distributed in all treating hospitals, both public and private
4. **Policy**
  - Advocacy toward making SBE a notified disease
  - Advocacy for bringing SBE under the National health schemes
  - Advocacy for removing SBE from the MLC (Medicolegal case) list
  - Strengthening the National Program for Control and prevention of snakebite
  - Guidelines for the procurement of venom for research purposes in order to facilitate research
5. **Research**
  - Evidence generation on the incidence, mortality, morbidity, and socioeconomic burden related to SBE in India
  - Estimating Disability Weights (DW) for snakebite
  - Venom characterization of medically relevant species other than for the Big 4
  - Development of point-of-care (POC) diagnostic devices for determining snakebite envenomation
  - Developing a Coding system for snakebite based on the syndromic approach
6. **Preventive Measures** [14,15]
  - Creating simple audio-visual modules in the local language about snake bite prevention and first-aid measures if bitten
  - Training of frontline health workers on first-aid measures and sentinel signs and symptoms of envenoming
  - Including a chapter on snakes and prevention in the Environmental Science textbook in schools
7. **One-health approach to SBE** [7]
  - An often-missed point is that SBE is a frequent cause of the loss of livestock in India. Liaising with veterinarians, ophiologists, and environmental scientists on a one-health approach to SBE and its prevention
8. **Mapping of hotspots digitally**
  - Mapping of hotpots based on the density of incidence and mortality related to SBE and on snake rescues from households or places of work
  - Building health system capacity for treatment in hot spots, as also ensuring adequacy of resources (beds, ventilators, human resources, ASV), especially during the snakebite season (onset of the monsoons).

## Possible roadblocks

The real challenge ahead is to bring all stakeholders from the domains of venom research, ophiology, and clinicians together on a common platform to work towards a common goal, under the auspices of the ICMR.

The other challenge is to contextualise IEC material to meet the diverse needs of India’s states and zones. The third major challenge is to sensitise politicians and policymakers about the issue and bring them on board.

## Funding

The ICMR which is the apex body in India for the formulation, coordination, and promotion of biomedical research, has designated 25 centres of excellence across specialities late 2023. The identified centres are not funded as a centre, but are funded for projects undertaken or carried out. The mandate being for evidence creation for which projects are funded on a case-to-case basis and not through the process of the standard routine call-for-proposals.

In summary, the team from the CCoE would be funded on projects (on merit) on a standalone basis. The funding would be from the ICMR-DHR (Department of Health research) for the proposals/projects submitted. The current administrative head of the ICMR seems very committed to the cause of snakebite.

AIMS the designated CCoE have just completed the National study on the incidence, mortality, morbidity, and economic burden of snakebite, which covered 14 states (of the 26) and between 2 to 4 districts in each state. The other project currently completed as a CCoE is on a modification to the age-old “snakes and ladders” game contextualised to region/geography for the sake of creating awareness on common venomous species typical to the region. The second project submitted as a CCoE is on the ICD coding for medically relevant snakes of India.

The CCoE in the future would serve as a repository for evidence in the domain of snakebite and SBE, helping coordinate and direct research, generate evidence and liaise with polity and policymakers towards mitigation and prevention. The CCoE would align activities with the WHO-SDG goal of reducing mortality by 50% by the year 2030.
